# Combining Polymerization and Templating toward Hyper-Cross-Linked Poly(propargyl aldehyde)s and Poly(propargyl alcohol)s for Reversible H_2_O and CO_2_ Capture and Construction of Porous Chiral Networks

**DOI:** 10.3390/polym15030743

**Published:** 2023-02-01

**Authors:** Lucie Havelková, Bogdana Bashta, Alena Hašková, Alice Vagenknechtová, Eliška Vyskočilová, Jiří Brus, Jan Sedláček

**Affiliations:** 1 Department of Physical and Macromolecular Chemistry, Faculty of Science, Charles University, Hlavova 2030, 128 43 Prague, Czech Republic; 2 Department of Gaseous and Solid Fuels and Air Protection, University of Chemistry and Technology in Prague, Technická 5, 166 28 Prague, Czech Republic; 3 Department of Organic Technology, University of Chemistry and Technology in Prague, Technická 5, 166 28 Prague, Czech Republic; 4 Institute of Macromolecular Chemistry, Czech Academy of Sciences, Heyrovský Sq. 2, 162 00 Prague, Czech Republic

**Keywords:** porous polymers, hyper-cross-linked, polyacetylenes, chiral modification, water harvesting

## Abstract

Two series of hyper-cross-linked microporous polyacetylene networks containing either -[CH=C(CH=O)]- or -[CH=C(CH_2_OH)]- monomeric units are reported. Networks are prepared by chain-growth copolymerization of acetal-protected propargyl aldehyde and acetal-protected propargyl alcohol with a 1,3,5-triethynylbenzene cross-linker followed by hydrolytic deprotection/detemplating. Deprotection not only liberates reactive CH=O and CH_2_OH groups in the networks but also modifies the texture of the networks towards higher microporosity and higher specific surface area. The final networks with CH=O and CH_2_OH groups attached directly to the polyene main chains of the networks have a specific surface area from 400 to 800 m^2^/g and contain functional groups in a high amount, up to 9.6 mmol/g. The CH=O and CH_2_OH groups in the networks serve as active centres for the reversible capture of CO_2_ and water vapour. The water vapour capture capacities of the networks (up to 445 mg/g at 297 K) are among the highest values reported for porous polymers, making these materials promising for cyclic water harvesting from the air. Covalent modification of the networks with (*R*)-(+)-3-aminopyrrolidine and (*S*)-(+)-2-methylbutyric acid enables the preparation of porous chiral networks and shows networks with CH=O and CH_2_OH groups as reactive supports suitable for the anchoring of various functional molecules.

## 1. Introduction

Organic polymer networks with permanent microporosity and high specific surface area, often referred to as porous organic polymers (POP) [1,2], represent an intensively studied group of porous materials, promising for catalytic [3,4], sorption [5,6,7], separation [8,9], sensing [10], and other applications. The high application potential of POPs reflects the wide variability of texture, composition, and covalent structure that can be achieved with these materials by properly combining and tuning the polymer and organic synthesis procedures used for their preparation. For example, POPs containing various acid and base groups, covalently inbuilt organometallic complexes, and electroactive and photoactive segments were developed for applications in heterogeneous organocatalysis [11,12], organometallic catalysis [13], electrocatalysis [14], and photocatalysis [15]. Texturally and covalently diverse POPs were prepared for reversible CO_2_ capture: non-functional POPs with an ultra-high specific surface area were revealed to be efficient for high-pressure CO_2_ capture [16], while functionalized POPs with a significantly lower specific surface area were suitable for the low-pressure CO_2_ trapping [17,18]. Several POPs with polar segments were prepared and tested in reversible water vapour capture from the air [19,20]. On the contrary, highly hydrophobic fluorine-rich POPs were also prepared [21,22] and applied to capture the vapour of non-polar hydrocarbons and fluorinated compounds. Application-interesting POPs with ionic [20,23], luminescent [24], chiral [25,26], and other groups [27,28] were also reported in the literature. Unlike inorganic porous materials (zeolites, molecular sieves) or MOF-type materials, POPs are mostly highly stable in an aqueous environment, even in the presence of acids, bases, and salts. Thanks to this, POPs appear to be promising adsorbents for the capture of a wide range of adsorptives and are comparable to COF-type adsorbents.

Functionalization of POPs (crucial for most applications) was mostly achieved by the so-called prepolymerization functionalization approach consisting of the polymerization of monomers bearing heteroatom groups or segments in addition to the polymerization-active groups. Bivalent and trivalent heteroatom segments were inbuilt into POPs, often in the form of networks knots created in the course of polymerization (e.g., triazole [29], imine knots [30]) or in the form of cyclic building blocks [17,31,32,33,34]. Univalent functional groups were inbuilt into POPs mostly as substituents (pendant groups) of hydrocarbon aromatic building blocks. For example, Cooper et al. copolymerized 1,3,5-triethynylbenzene with dibromoarenes ring-substituted with various groups (NO_2_, OMe, COOMe, CF_3_, NH_2_, OH) via Sonogashira cross-coupling under elimination of HBr and formation of functionalized poly(aryleneethynylene) type POPs. The functional groups were preserved in the resulting POPs [35]. This concept was adopted by many other authors, and POPs with various univalent functional groups were prepared by step-growth polymerizations using coupling [36], cyclotrimerization [37], knitting [36,38], and other reactions. The chain-growth polymerization approach was also efficient for the synthesis of functionalized POPs. Radical polymerizations of vinyl comonomers and cross-linkers into functionalized POPs were well reviewed in ref. [39]. Our group described the preparation of functionalized polyacetylene POPs by chain-growth coordination and spontaneous polymerizations of ring-functionalized arylacetylenes [9,40,41].

The microporosity of POP is created during polymerization due to the rigidity of the building blocks of POPs and their extensive cross-linking, which prevents tight packing of the network segments. As a result, POPs contain unoccupied space forming micropores. Micropores can be accompanied by larger mesopores in POPs. Mesopores are believed to be formed by the covalent interconnecting of small particles of the microporous network in the later stages of polymerization. In some cases, the porosity of POPs was successfully modified by templating approach. Seo et al. used readily hydrolyzable polylactides as a template to introduce mesopores into hyper-cross-linked POPs prepared by a combination of radical polymerization and knitting reaction [42,43]. A combination of polymerization and high internal phase emulsion templating was efficient for preparing POPs containing both micropores and macropores [44,45]. Huang et al. used radical copolymerization of mixtures of monomers (di-*tert*-butyl-4,4′-stilbene dicarboxylate, *tert*-butyl 4-maleimidobenzoate, *tert*-butyl 4-vinylbenzoate, and divinylbenzene cross-linker) to prepare microporous POPs. The postpolymerization thermal or chemical modification led to the removal of *tert*-butyl groups accompanied by an increase in the micropore volume and the specific surface area of these POPs [46]. We recently reported hyper-cross-linked polyacetylene networks with aromatic template segments covalently attached to the scaffold via azomethine links. The postpolymerization hydrolysis of these links and the removal of template molecules led to a significant modification of the micropore size distribution and the specific surface area of the POPs [47,48]. In some cases, even non-porous networks were modified into POPs with a specific surface area of about 500 m^2^/g by this templating approach [48].

In this paper, we report POPs of hyper-cross-linked polyacetylene-type with CH=O and CH_2_OH groups attached directly (without any spacer) to the rigid polyacetylene main chains. Acetal-protected aliphatic monomers and chain-growth copolymerization followed by deprotection and detemplating were used for the synthesis. Prepared POPs with a tuneable content of functional groups and texture were highly active in reversible water vapour capture and were demonstrated as supports for covalent anchoring of various functional molecules, e.g., chiral molecules, under preservation of porosity. The functional properties of POPs are discussed in relation to pore size distribution and the content of functional groups.

## 2. Materials and Methods

### 2.1. Materials

(Acetylacetonato)(norbornadiene)rhodium(I), [Rh(nbd)acac], (>98%), 1,3,5-triethynylbenzene, (TEB) (98%), propargylaldehyde diethyl acetal, (M1) (>97%), pent-1-yne (>98%) (all TCI Europe N.N., Zwijndrecht, Belgium), and acetaldehyde ethyl propargyl acetal, (M2) (98%, Acros Organics, Geel, Belgium) were used as obtained. Dichloromethane (99.95%, Lach-Ner Ltd., Neratovice, Czech Republic) was distilled with P_2_O_5_.

The following chemicals for the modifications were used as received: dansyl hydrazine (>97%), (*R*)-(+)-3-aminopyrrolidine (>98%) (all TCI Europe N.N., Zwijndrecht, Belgium), (*S*)-(+)-2-methylbutyric acid (98%, Sigma Aldrich Ltd., Prague, Czech Republic), *p*-toluenesulfonic acid (monohydrate, p.a., Lachema, Neratovice, Czech Republic), methanol (99.99% Lach-Ner Ltd., Neratovice, Czech Republic), benzene (anhydrous, 99.8%, Sigma Aldrich Ltd., Prague, Czech Republic).

### 2.2. Copolymerization

Copolymerizations of protected monomers M1 and M2 with 1,3,5-triethynylbenzene (TEB) were performed in CH_2_Cl_2_ at 75 °C in a sealed thick-wall ampoule under an argon atmosphere using [Rh(nbd)acac] as a polymerization catalyst. The overall initial concentration of monomers was 0.3 mol/dm^3^; the concentration of [Rh(nbd)acac] was 15 mmol/dm^3^. The mole ratios of the comonomers in the copolymerization feed were either M1 or M2:TEB = 1:1 for P(M1/TEB 1:1) and P(M2/TEB 1:1) networks, respectively, or M1 or M2:TEB = 3:1 concerning P(M1/TEB 3:1) and P(M2/TEB 3:1) networks, respectively. The polymerizations were started by adding the catalyst solution to the solution of monomers. The polymerizations were finished after 7 days by diluting the reaction mixture (containing the solid copolymer) with an excess of CH_2_Cl_2_. The solid copolymer network was separated, repeatedly washed with CH_2_Cl_2_, dried under vacuum at room temperature for 2 days to constant weight, and finally mechanically ground. The yield was determined gravimetrically. All polymerizations proceeded with quantitative yield.

### 2.3. Deprotection of Parent Networks

Typically, 400 mg of the parent copolymer network with protecting acetal groups were kept under stirring in deionized water (100 mL) containing HCl for up to 2 weeks at room temperature. The mole ratio of HCl to protecting groups was 10:1. Then the detemplated copolymer product was thoroughly washed to neutral pH with deionized water and dried under vacuum for 2 days at room temperature.

Details on the postpolymerization modification of P(M1/TEB 1:1)H and P(M2/TEB 1:1)H are given in Supplementary Material.

### 2.4. Techniques

#### 2.4.1. NMR

All ^13^C cross-polarization magic angle spinning (CP/MAS) NMR spectra were measured at 11.7 T using a Bruker Avance III 500 WB/US NMR spectrometer (Bruker Corporation, Billerica, MA, USA), as described elsewhere [17].

#### 2.4.2. N_2_ and CO_2_ Adsorption

The adsorption/desorption isotherms of nitrogen (at 77 K) and CO_2_ (at a range from 273 to 333 K) were measured at a Triflex V4.02 apparatus (Micromeritics Instrument Corporation, Norcross, GA, USA). Prior to the sorption measurements, all the samples were degassed using Micromeritics SmartVacPrep instrument as described previously [17].

The Brunauer–Emmett–Teller specific surface area, *S*_BET_, total pore volume, *V*_tot_, and micropore volume *V*_mi_ are reported. The *V*_mi_ and *V*_tot_ values were determined according to the N_2_ amount trapped at *p*/*p*_0_ = 0.1 and *p*/*p*_0_ = 0.97, respectively. The micropore size distribution was determined by the semi-empirical method of Horvath–Kawazoe. A model for the slit pore geometry (original Horvath–Kawazoe) with carbon–graphite adsorbent was used for the calculation of the size of the pores.

#### 2.4.3. TGA

Thermogravimetric analysis (TGA) was performed on the Setsys Evolution apparatus (Seteram, Caluire-et-Cuire, France) under a nitrogen atmosphere using a heating rate 10 °C/min in a temperature range from 40 to 800 °C.

#### 2.4.4. DVS

The water vapour sorption was measured using a DVS Advantage 2 instrument (Surface Measurement Systems Ltd., London, UK) at 297 K. The device allows monitoring of moisture adsorption under specified conditions.

Approximately 25 mg of the polymer network was first preheated to 100 °C for 120 min. The adsorption/desorption analysis was performed with a relative humidity (RH) setting of 0%–90%–0%–90%–0% (with intermediate steps of 10% RH) and a setting of 180 min for each step.

#### 2.4.5. SEM

SEM measurements were performed using Tescan Lyra3 apparatus (TESCAN Brno, Ltd., Brno, Czech Republic) at an accelerating voltage of 10 kV.

## 3. Results and Discussion

### 3.1. Synthesis and Characterization of the Porous Networks

**Scheme 1:**
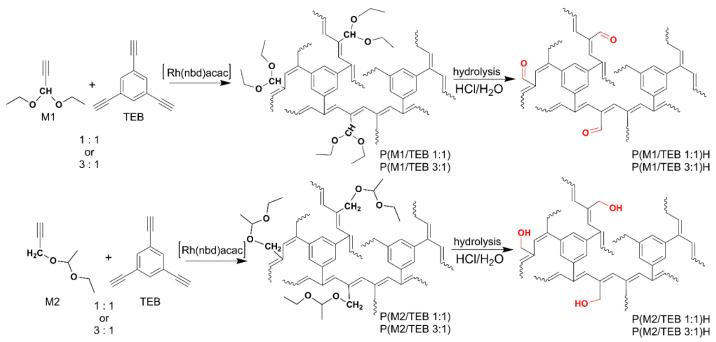
Preparation of porous networks with CH=O and CH_2_OH groups via chain-growth polymerization followed by hydrolytic deprotection.

The synthetic part of this study aimed to prepare porous hyper-cross-linked polyacetylene networks containing carbaldehyde (-CH=O) and hydroxymethyl (-CH_2_OH) groups attached (without any spacer) to the carbon atoms of linear vinylene units of the main chains of the networks. The chain-growth polymerization of substituted acetylenes used for the preparation of polyacetylenes is mostly reported to be tolerant to various heteroatom groups of the monomers [49,50,51]. However, in the case of the polyacetylene networks reported here, it was not possible to use respective acetylenes, prop-2-ynal, HC≡C-CH=O, and prop-2-yn-1-ol, HC≡C-CH_2_OH, as starting (co)monomers for their preparation. Prop-2-ynal (propargyl aldehyde) is an extremely unstable compound, as reported in the literature [52,53]. Although prop-2-yn-1-ol (propargyl alcohol) is a stable compound, it is also unsuitable for the preparation of polyacetylene networks. Our preliminary experiments showed a poor polymerizability of this compound, apparently due to the presence of a reactive OH group in the vicinity of the polymerization-active ethynyl group. For the preparation of the discussed networks, we, therefore, proposed (i) to use acetylene monomers with protected carbaldehyde and hydroxymethyl groups, (ii) to incorporate these monomers into networks by means of copolymerizations with a proper ethynylated cross-linker, and finally (iii) to deprotect these (parent) networks under the formation of the final products with desired -[CH=C(CH=O)]- and -[CH=C(CH_2_OH)]- monomeric units. Two monomers were used for this purpose (see Scheme 1): propargyl aldehyde diethyl acetal, M1 (i.e., the acetal-protected form of prop-2-ynal) and acetaldehyde ethyl propargyl acetal, M2 (i.e., the acetal-protected form of prop-2-yn-1-ol). Monomers M1 and M2 were (independently) copolymerized with 1,3,5-triethynylbenzene (TEB) serving as a cross-linker. Copolymerizations were catalysed with [Rh(nbd)acac] complex (see Scheme 1). The comonomer mole ratios in the feed, M1(or M2):TEB were 1:1 and 3:1 (see Section 2.2 for the details). All copolymerizations provided quantitative yields of respective networks, which were labelled as follows: P(M1/TEB 1:1), P(M1/TEB 3:1), P(M2/TEB 1:1), and P(M2/TEB 3:1). The prepared networks were dark brown solids, totally insoluble in water, methanol, dichloromethane, tetrahydrofuran, and benzene. All networks showed no observable swelling in any above solvents.

**Figure 1:**
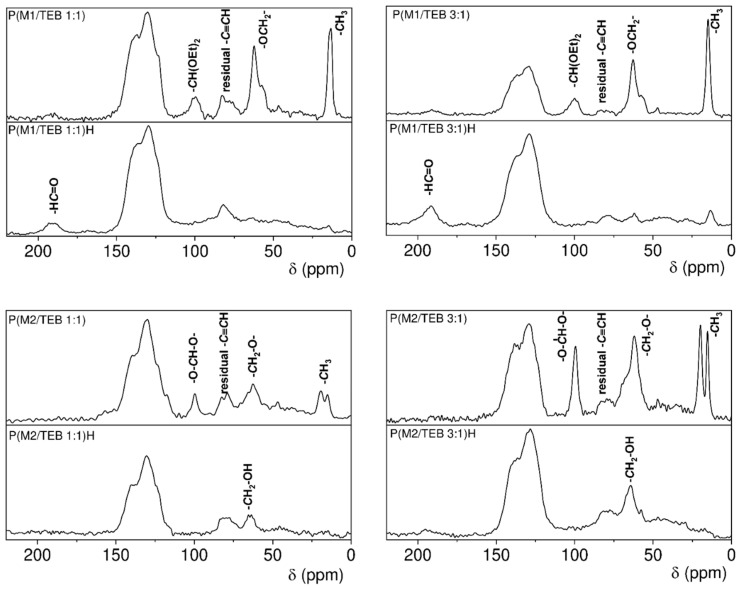
^13^C CP/MAS NMR spectra of prepared networks.

The ^13^C CP/MAS NMR spectra of P(M1/TEB 1:1), P(M1/TEB 3:1), P(M2/TEB 1:1), and P(M2/TEB 3:1) are shown in Figure 1. The broad dominating signal in the *δ* = 120–150 ppm region of all spectra corresponded to the resonance of aromatic carbon atoms of TEB units and polyene main-chain carbon atoms. The signal at *δ* = 80 ppm in all spectra was due to carbon atoms of unreacted ethynyl groups in the units formed from TEB. Evidently, not all TEB monomer molecules were incorporated into the networks through the polymerization transformation of all three ethynyl groups to form cross-linking units connecting the chains through benzene-1,3,5-triyl segments. Some TEB cross-linking units could contain one unreacted ethynyl group (cross-linking through benzene-1,3-diyl segments), and some TEB molecules could be incorporated into the networks as linear units possessing two unreacted ethyl groups (see ref. [54] for more details). The ^13^C CP/MAS NMR spectra of P(M1/TEB 1:1) and P(M1/TEB 3:1) contained the distinct signals of carbaldehyde diethyl acetal pendant groups of the M1-type monomeric units. The sharp signal at *δ* = 14 ppm corresponded to the carbon atoms of methyl groups, the signal at *δ* = 62 ppm corresponded to carbons of -O*C*H_2_- groups, and the signal at *δ* = 100 ppm was due to the -*C*H(OEt)_2_ carbon atoms. Similarly, the ^13^C CP/MAS NMR spectra of P(M2/TEB 1:1) and P(M2/TEB 3:1) contained signals characteristic of acetaldehyde ethyl methyl acetal pendant groups of the M2-type monomeric units. Partly distinguished signals at *δ* = 15 ppm and *δ* = 20 ppm belonged to carbon atoms of two different methyl groups of M2-type monomeric units (see Scheme 1). The signal at *δ* = 62 ppm corresponded to the resonance of carbon atoms of -O*C*H_2_- groups and the signal at *δ* = 100 ppm was due to the carbon atoms of -O-*C*H(CH_3_*)*-O- groups. As evident from Figure 1, the signals of carbon atoms of acetal groups were more pronounced in the spectra of P(M1/TEB 3:1), and P(M2/TEB 3:1) than in the spectra of P(M1/TEB 1:1), and P(M2/TEB 1:1). This corresponded well with the fact that P(M1/TEB 3:1), and P(M2/TEB 3:1) were richer in acetal groups containing units than P(M1/TEB 1:1) and P(M2/TEB 1:1). The above discussed ^13^C CP/MAS NMR spectra of P(M1/TEB 1:1), P(M1/TEB 3:1), P(M2/TEB 1:1), and P(M2/TEB 3:1) networks were in good agreement with the structure of the networks given in Scheme 1. Prepared polymer networks consisted of polyacetylene (polyvinylene) main chains cross-linked with benzene linkers formed from TEB. The linear units of the networks were decorated with acetal-protected carbaldehyde or hydroxymethyl groups. The spectra confirmed that no significant deprotection (detectable by ^13^C CP/MAS NMR spectroscopy) occurred during the synthesis of the networks.

Networks P(M1/TEB 1:1), P(M1/TEB 3:1), P(M2/TEB 1:1), and P(M2/TEB 3:1) were subsequently submitted to the hydrolysis in HCl/H_2_O (for details see Section 2.3) in order to decompose their acetal pendant groups and remove the low-molecular-weight products of the hydrolysis from the networks. Optimization of the hydrolysis procedure showed that a long reaction time (two weeks at room temperature) was required to ensure the quantitative extent of this process. The hydrolytic deprotection resulted in polymer networks labelled as follows: P(M1/TEB 1:1)H, P(M1/TEB 3:1)H, P(M2/TEB 1:1)H, and P(M2/TEB 3:1)H. ^13^C CP/MAS NMR spectroscopy confirmed that the hydrolytic deprotection was highly effective, both in the case of networks with M1-type units and in the case of networks with M2-type units. This is evident from Figure 1, in which the ^13^C CP/MAS NMR spectra of hydrolyzed networks P(M1/TEB 1:1)H, P(M1/TEB 3:1)H, P(M2/TEB 1:1)H, and P(M2/TEB 3:1)H are compared with the ^13^C CP/MAS NMR spectra of parent networks P(M1/TEB 1:1), P(M1/TEB 3:1), P(M2/TEB 1:1), and P(M2/TEB 3:1). The ^13^C CP/MAS NMR signals of aliphatic carbon atoms of acetal protecting groups were either absent or very weak in the spectra of all hydrolyzed networks. At the same time, ^13^C CP/MAS NMR spectroscopy confirmed that the hydrolysis of the parent networks resulted in networks with the required carbaldehyde and hydroxymethyl groups. The ^13^C CP/MAS NMR spectra of P(M1/TEB 1:1)H and P(M1/TEB 3:1)H contained a well-resolved signal of carbon atoms of -CH=O groups at *δ* = 190 ppm. The presence of CH_2_OH groups in P(M2/TEB 1:1)H and P(M2/TEB 3:1)H was manifested by a signal at *δ* = 62 ppm in ^13^C CP/MAS NMR spectra (see Figure 1).

**Figure 2:**
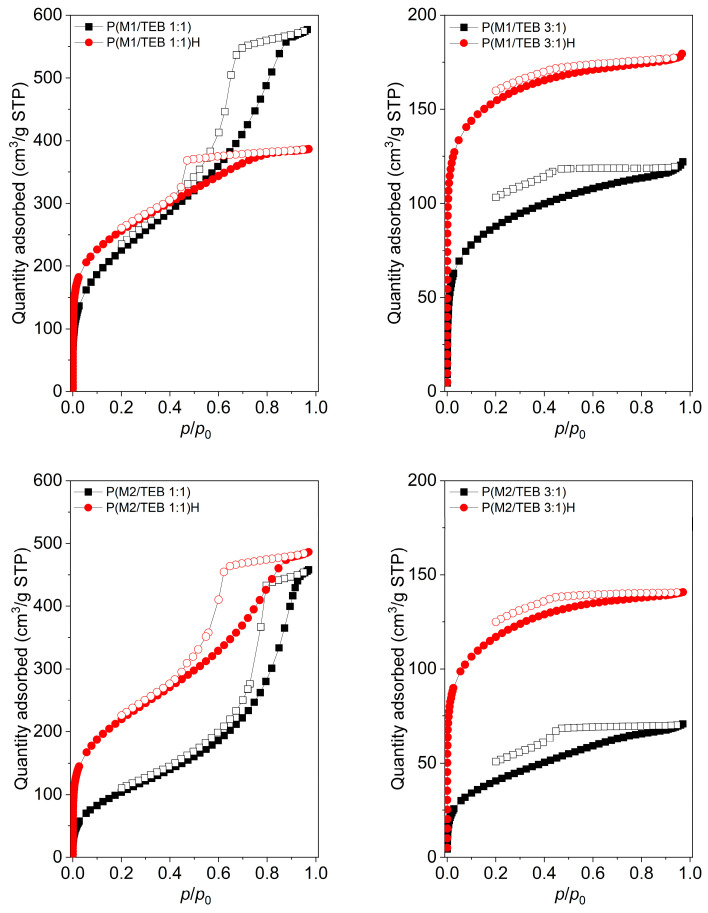
N_2_ adsorption (full points) and desorption (empty points) isotherms (77 K) on the prepared networks.

**Figure 3:**
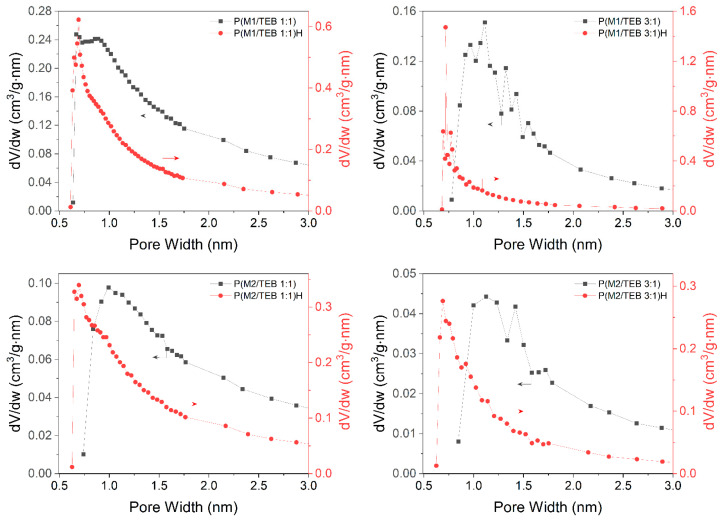
Micropore size distribution of prepared networks.

**Figure 4:**
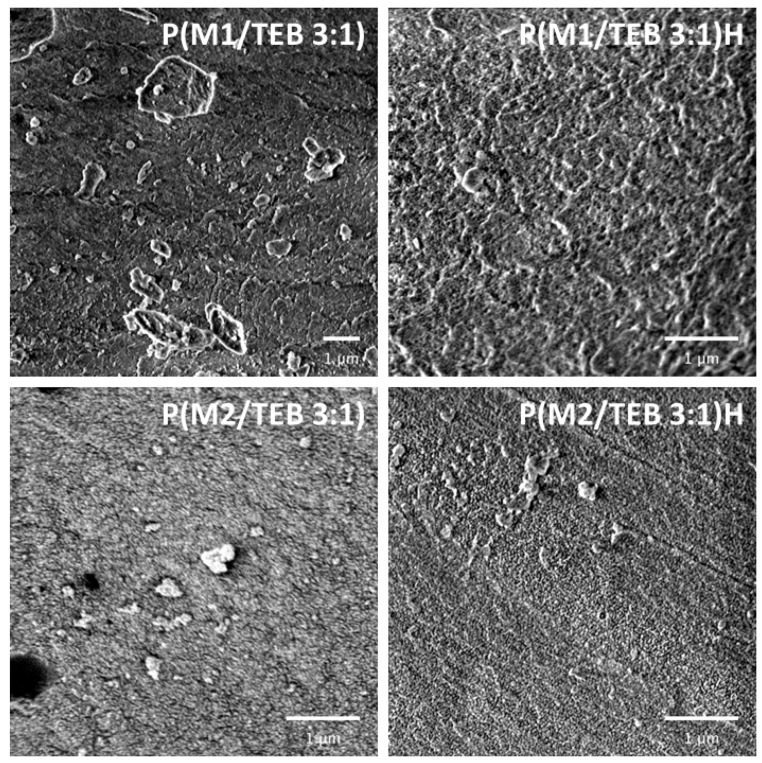
SEM images of the parent and hydrolyzed networks.

The covalent structure of the studied networks (rigid polyene main chains hyper-cross-linked with rigid aromatic linkers) was designed so that these networks could exhibit permanent porosity. The nitrogen adsorption/desorption measurements at 77 K confirmed this assumption: both parent and hydrolyzed networks exhibited a micro/mesoporous texture, as evident from the N_2_ adsorption/desorption isotherms shown in Figure 2. The presence of the micropores in the networks was manifested by sharp N_2_ uptake at low relative pressures. An increase in the adsorbed amount of N_2_ at higher relative pressures and a hysteresis on the adsorption/desorption isotherms indicated the presence of mesopores and/or interparticle void volume in the networks. The values of texture parameters of the networks ascertained from the N_2_ adsorption isotherms, i.e., Brunauer–Emmett–Teller specific surface area (*S*_BET_), micropore volume (*V*_mi_), and total pore volume (*V*_tot_), are summarized in Table 1. The texture parameters of the parent networks depended on the type and content of linear units. The values of *S*_BET_, *V*_mi_, and *V*_tot_ decreased with increasing content of the M1- and M2-type units in the networks. It is obvious that the pendant groups of M1- and M2-type units, due to their flexibility, did not contribute to the formation of porosity and, on the contrary, could partly occupy the potential pores in the networks. Nevertheless, even in the case of P(M1/TEB 3:1) and P(M2/TEB 3:1), micro/mesoporosity and *S*_BET_ of 314 and 149 m^2^/g, respectively, were achieved. Parent networks containing M1-type linear monomeric units generally showed higher values of *S*_BET_, *V*_mi_, and *V*_tot_ than parent networks with M2-type linear monomeric units. For example, the *S*_BET_ of 832 m^2^/g was achieved for P(M1/TEB 1:1) while P(M2/TEB 1:1) exhibited a specific surface area roughly 50% lower (*S*_BET_ = 393 m^2^/g). The reason could be the higher symmetry of the pendant groups of the M1-type units, thanks to which these groups might occupy less space in the network. The N_2_ adsorption/desorption measurements further clearly proved that the above-described hydrolytic deprotection of M1- and M2-type linear units of parent networks was accompanied by an increase in the *S*_BET_ and *V*_mi_ values of the networks. All the hydrolyzed networks, i.e., P(M1/TEB 1:1)H, P(M1/TEB 3:1)H, P(M2/TEB 1:1)H, and P(M2/TEB 3:1)H had higher *S*_BET_ and *V*_mi_ values than their parent counterparts. (see Table 1 and Figure 2). The increase in *S*_BET_ due to hydrolytic deprotection was particularly significant in the case of networks containing M2-type units: compared to the parent networks P(M2/TEB 1:1) and P(M2/TEB 3:1), the hydrolyzed networks P(M2/TEB 1:1)H and P(M2/TEB 3:1)H showed more than twice higher specific surface area values (Table 1). The increase in the micropore volume due to the hydrolysis was also significant in these networks. Parent networks P(M2/TEB 1:1) and P(M2/TEB 3:1) showed *V*_mi_ of 0.13 and 0.05 cm^3^/g, respectively, while the *V*_mi_ values of their hydrolyzed counterparts were 0.29 cm^3^/g (P(M2/TEB 1:1)) and 0.17 cm^3^/g (P(M2/TEB 3:1)). Moreover, the hydrolysis of the networks containing M1-type units was accompanied by an increase in *S*_BET_ and *V*_mi_ value although this increase was less pronounced. The *S*_BET_ values increased from 832 to 911 m^2^/g due to the transformation of P(M1/TEB 1:1) to P(M1/TEB 1:1)H, the transformation of P(M1/TEB 3:1) to P(M1/TEB 3:1)H was accompanied by an increase in *S*_BET_ from 313 to 534 m^2^/g. Figure 3 compares the micropore size distributions of the parent and hydrolyzed networks. The values of micropore width corresponding to the maxima of the distributions (*D*_mi_) are given in Table 1. In the case of all networks, the micropore size distribution was shifted towards lower values of micropore width as a result of hydrolytic deprotection. The *D*_mi_ values of the parent networks P(M1/TEB 1:1), P(M1/TEB 3:1), P(M2/TEB 1:1), and P(M2/TEB 3:1) ranged from 0.9 to 1.1 nm while *D*_mi_ = 0.7 nm was determined for all hydrolyzed networks (Table 1). The hydrolytic deprotection of the parent networks proceeded through the decomposition of acetal groups, followed by the removal of aliphatic low-molecular-weight hydrolytic products from the networks. It should be noted that this process was not accompanied by a collapse of the hyper-cross-linked scaffold of the networks. On the contrary, the void volume occupied by the aliphatic protecting groups in the parent networks was emptied under the formation of new micropores in the hydrolyzed networks. This led in parallel to an increase in *S*_BET_ values. Figure 4 shows scanning electron microscopy (SEM) images of the parent and hydrolyzed networks. The SEM method did not show any morphological changes due to the deprotection of the networks. Figure S1 of the Supplementary Materials shows the results of thermogravimetric analysis (TGA). The weight loss of all deprotected networks was less than 2% at 200 °C. The temperature values at which a weight loss of 5% was detected were as follows: 241 °C, P(M1/TEB 3:1)H, 248 °C, P(M2/TEB 3:1)H, 264 °C, P(M1/TEB 1:1)H, and 275 °C P(M2/TEB 1:1)H. Thus, the hydrolyzed networks showed relatively high temperature stability.

As discussed above, the hydrolytic deprotection of the parent networks was primarily aimed at obtaining networks with reactive carbaldehyde and hydroxymethyl groups. From the point of view of the texture of the networks, the performed deprotection can be considered at the same time as a detemplating process positively modifying the texture of the networks. The applied synthesis combining chain polymerization and deprotection/detemplating thus enabled the preparation of porous networks with a high content of CH=O and CH_2_OH groups (up to 9.6 mmol/g) and a satisfactory specific surface area (*S*_BET_ from 400 to 800 m^2^/g) suitable for various applications, as described in the following subsections.

### 3.2. Water Vapour and Carbon Dioxide Capture

In order to evaluate the effect of CH=O and CH_2_OH groups of P(M1/TEB 1:1)H, P(M1/TEB 3:1)H, P(M2/TEB 1:1)H and P(M2/TEB 3:1)H on the efficiency of these networks in capturing H_2_O and CO_2_ a heteroatom-free network was prepared as a comparison sample. Copolymerization of 1-pentyne with TEB cross-linker (3:1 in the feed) provided a P(pentyne/TEB 3:1) network containing -[CH=C(CH_2_CH_2_CH_3_)]- linear units and showing the following texture parameters: *S*_BET_ = 810 m^2^/g, *V*_mi_ = 0.28 cm^3^/g, and *V*_tot_ = 1.69 cm^3^/g. Details on the synthesis and characterization of P(pentyne/TEB 3:1) are given in Supplementary Materials (Scheme S1, Table S1, Figures S2–S4).

**Scheme 2:**
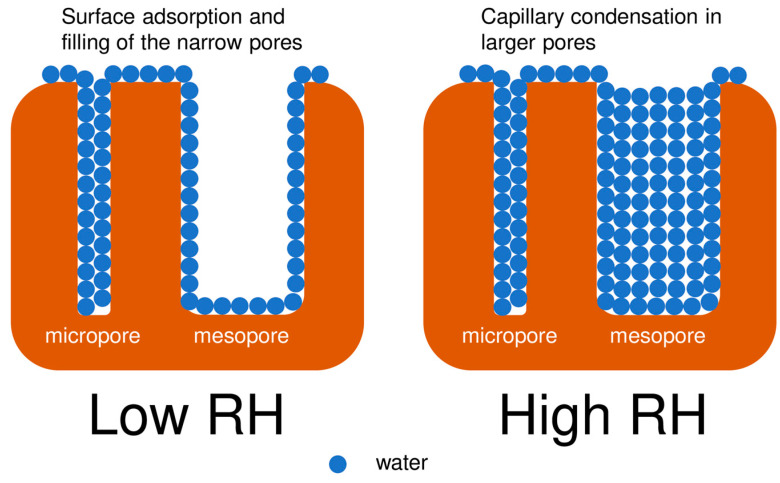
Schematic mechanism of H_2_O capture in networks.

**Figure 5:**
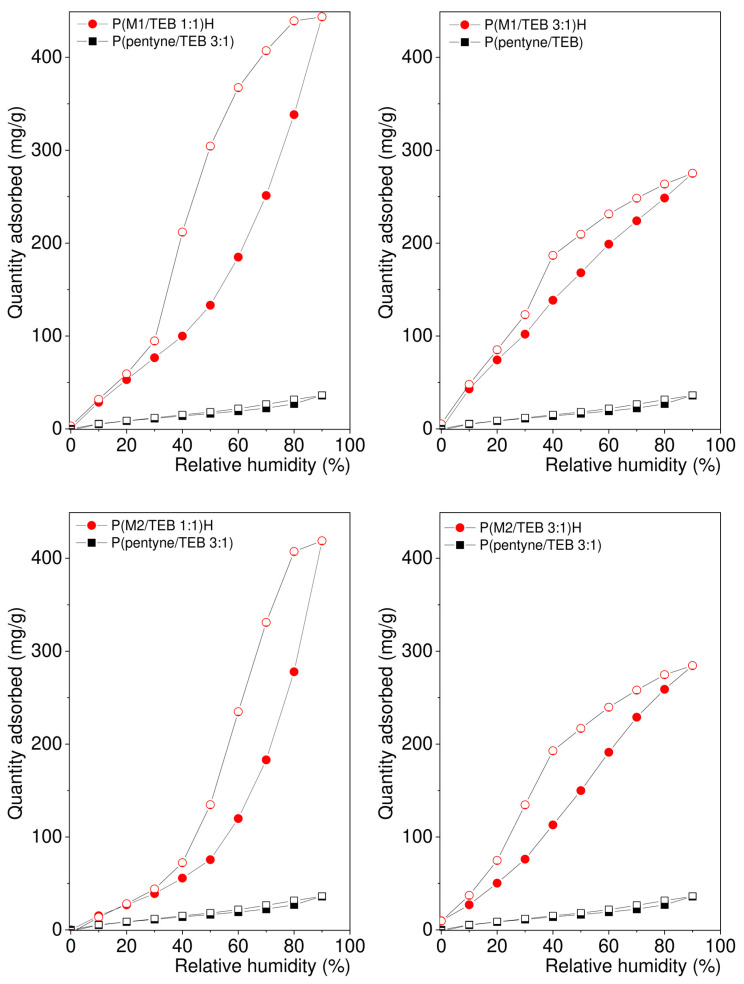
Water adsorption (full points) and desorption (empty points) isotherms (297 K) on the prepared networks.

The efficiency of the networks in capturing and releasing water vapour was investigated at 297 K (see Section 2.4.4 for detail). The H_2_O adsorption/desorption isotherms are given in Figure 5. The corresponding time course of H_2_O adsorption/desorption is shown in Figure S5 of the Supplementary Materials. Table 2 summarizes the capture capacities of the networks for H_2_O (i) at relative humidity (RH) of 90% (*a*_H2ORH90_) and (ii) at RH = 40% (*a*_H2ORH40_). It is evident that the polar CH=O and CH_2_OH groups in the prepared networks dramatically increased the efficiency of these materials in capturing water vapour in comparison to the hydrocarbon network P(pentyne/TEB 3:1). The values of *a*_H2ORH90_ ranged from 272 to 445 mg/g for CH=O and CH_2_OH groups containing networks while *a*_H2ORH90_ was only 36 mg/g for P(pentyne/TEB 3:1). The data in Table 2 and the character of the isotherms in Figure 5 indicated that two processes were most likely involved in the capture of water vapour in the functionalized networks: (i) adsorption of H_2_O molecules on the surface of pores accompanied by trapping of H_2_O in narrow micropores and (ii) capillary condensation of water in larger pores [55] (see Scheme 2). At lower RH values, the adsorption and filling of narrow micropores prevailed. The efficiency of these processes increased with the increasing content of polar groups in the networks. This is evident from Table 2, showing an increase in the value of *a*_H2ORH40_ in parallel with an increase in the content of CH=O and CH_2_OH groups in the networks in the following series: P(M1/TEB 1:1)H ˂ P(M1/TEB 3:1)H and P(M2/TEB 1:1)H ˂ P(M2/TEB 3:1)H. We assume that the CH=O and CH_2_OH groups served in the networks as adsorption centres interacting with H_2_O molecules via dipole–dipole interactions or weak hydrogen bonding. At higher RH values, the process of capillary condensation of H_2_O in the larger pores most probably prevailed and thus significantly contributed to the values of capture capacities reached when RH = 90%, *a*_H2ORH90_. The values of *a*_H2ORH90_ increased in the series: P(M1/TEB 3:1)H ˂ P(M1/TEB 1:1)H and P(M2/TEB 3:1)H ˂ P(M2/TEB 1:1)H. This increase did not follow an increase in the extent of functionalization of the networks, but it correlated with an increase in *V*_tot_ values (see Table 2). We assume that the size of the pore volume available for capillary condensation (volume localized in mesopores or larger micropores) was an important or decisive factor in the efficiency of this process [56]. It is worth noting that in the case of P(M1/TEB 3:1)H and P(M2/TEB 3:1)H, the volume of water captured when RH = 90% roughly corresponded to the total pore volume of these networks determined by N_2_ adsorption (see Table 2). It should, however, be emphasized that water capture by capillary condensation did not occur with the hydrocarbon network P(pentyne/TEB 3:1), despite its high *V*_tot_ value (1.69 cm^3^/g). For effective capture of water by capillary condensation, prior coverage of the pore surface with water trapped by adsorption was evidently necessary.

The participation of capillary condensation in water capture on functionalized networks corresponded with the hysteresis loops on the adsorption/desorption isotherms given in Figure 5. The hysteresis loops were closed in the case of P(M1/TEB 1:1)H, P(M1/TEB 3:1)H, and P(M2/TEB 1:1)H when RH ≈ 20%, and the complete desorption of water from these networks was achieved by isothermal reduction of RH without the need to increase the temperature. In the case of network P(M2/TEB 3:1)H, the hysteresis loop did not completely close even when RH = 0% (equilibration time 180 min). Nevertheless, the amount of water remaining in the network under these conditions was only 9 mg/g. The water adsorption/desorption isotherms on functionalized networks were well reproducible, as evident from Figure S6 in Supplementary Materials showing the isotherms for two consecutive H_2_O adsorption/desorption cycles. Water capture capacities when RH = 90% of P(M1/TEB 1:1)H and P(M2/TEB 1:1)H, *a*_H2ORH90_ = 445 mg/g and *a*_H2ORH90_ = 422 mg/g, respectively, corresponded to the highest values reported for sorbents of the porous polymer-type [19,55]. For example, polyphenylene-type POPs functionalized with NH_2_ and NO_2_ groups exhibited water capture capacities from 290 to 350 mg/g (RH = 97%, 297 K) [37]. Values of *a*_H2ORH90_ up to 420 mg/g (297 K) were achieved on POPs with epoxy functional groups [57]. Pyridine containing hyper-cross-linked POPs reported recently by our group exhibited *a*_H2ORH90_ up to 376 mg/g [17]. Water capture capacity of about 550 mg/g, (RH = 90%, 297 K) was reported for ionic POPs with quaternized 1,4-diazabicyclo[2.2.2]octane-type units [20]. Water capture capacities of P(M1/TEB 1:1)H and P(M2/TEB 1:1)H were comparable with capacities reported for various COF-type materials (e.g., COFs with functionalized aromatic building blocks, *a*_H2ORH90_ up to 700 mg/g [58] and COFs with imine links, *a*_H2ORH90_ up to 300 mg/g [59]). However, it should be mentioned that COFs showed water capture activity only at higher RH values (mostly RH > 30%). The final synthesis stage of P(M1/TEB 1:1)H and P(M2/TEB 1:1)H was carried out in an aqueous environment, which confirms the high resistance of these materials to water. P(M1/TEB 1:1)H and P(M2/TEB 1:1)H networks are, therefore, more suitable for repeated water capture than porous materials of the zeolite or MOF types, which can degrade during long-term contact with water. The good reproducibility of water vapour capture and the high *a*_H2ORH90_ values achieved for P(M1/TEB 1:1)H and P(M2/TEB 1:1)H make these networks promising for so-called cyclic water harvesting from the air [60]. Assume that samples P(M1/TEB 1:1)H and P(M2/TEB 1:1)H would work isothermally at 297 K between RH = 20% and RH = 80% in this process. The amount of reversibly trapped/released water would then be 280 mg/g for P(M1/TEB 1:1)H and 250 mg/g for P(M2/TEB 1:1)H.

The efficiency of the networks in capturing and releasing CO_2_ was investigated by means of adsorption/desorption isotherms at 273, 293, 313, and 333 K (see Figure S7, Supplementary Materials). The CO_2_ capture on all networks was reversible at all temperatures tested (see Figure S7, Supplementary Materials). Table 3 summarizes the CO_2_ adsorption capacities (273 K) at an equilibrium CO_2_ pressure of 0.2 and 1 bar (values of *a*_CO2_ in mg(CO_2_)/g(network)). The *a*_CO2_ values (1 bar 273 K) of networks containing CH=O and CH_2_OH groups were roughly twice the *a*_CO2_ value resulting for hydrocarbon P(pentyne/TEB 3:1) network and corresponded to the capacities most frequently reported for CO_2_ capture on various functionalized porous polymers under these conditions [2,18]. The CO_2_ capture on the networks proceeded (under the conditions used) via the adsorption of CO_2_ molecules on the surface. As the individual networks had different specific surface areas, we also report (Table 3) the CO_2_ adsorption capacities related to 1 m^2^ of the network surface, *a*_CO2/S_ [*a*_CO2/S_ = *a*_CO2_/*S*_BET_].

**Figure 6:**
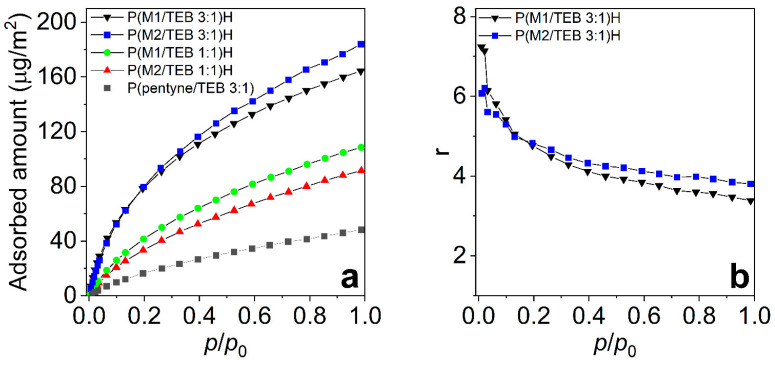
CO_2_ adsorption isotherms (273 K) on the networks (**a**), ratio *r* = *a*_CO2/S,P(M1/TEB3:1)H_/*a*_CO2/S,P(pentyne/TEB3:1)_ and *r* = *a*_CO2/S,P(M2/TEB3:1)H_/*a*_CO2/S,P(pentyne/TEB3:1)_ versus the equilibrium CO_2_ pressure (**b**).

Figure 6 shows the CO_2_ adsorption isotherms (*a*_CO2/S_ vs. the equilibrium CO_2_ pressure) at 273 K for all networks. The values of *a*_CO2/S_ increased significantly with increasing content of CH=O and CH_2_OH groups in the networks in the order: P(pentyne/TEB 3:1) ˂ P(M2/TEB 1:1)H~P(M1/TEB 1:1)H ˂ P(M1/TEB 3:1)H~P(M2/TEB 3:1)H. It is clearly visible that the CH=O and CH_2_OH groups significantly enhanced the efficiency of the networks in CO_2_ capture. The CH=O and CH_2_OH groups had a very similar effect, and the CO_2_ capture efficiency was influenced by the content of these groups rather than their type. To evaluate the influence of the CH=O and CH_2_OH groups of the networks on the efficiency of CO_2_ capture at different stages of this process, we used the ratios of the *a*_CO2/S_ values determined for P(M1/TEB 3:1)H and P(M2/TEB 3:1)H and the *a*_CO2/S_ values for P(pentyne/TEB 3:1) at the same CO_2_ pressure and at 273 K. This ratio was defined as *r* = *a*_CO2/S,P(M1/TEB3:1)H_/*a*_CO2/S,P(pentyne/TEB3:1)_ in the case of P(M1/TEB 3:1)H and *r* = *a*_CO2/S,P(M2/TEB3:1)H_/*a*_CO2/S,P(pentyne/TEB3:1)_ in the case of P(M2/TEB 3:1)H. The values of *r* were plotted versus equilibrium CO_2_ pressure in Figure 6. It is obvious that *r* decreased with increasing equilibrium CO_2_ pressure (i.e., with increasing coverage of the surface of the networks with CO_2_ molecules): while at a CO_2_ pressure of 0.05 bar, the *r* value was about 6, and it decreased to ~3.5 at a CO_2_ pressure of 1 bar. Most probably, CO_2_ molecules interacted with CH=O and CH_2_OH groups of enhanced affinity toward CO_2_ in the initial stage of adsorption. In the later stages of adsorption, CO_2_ was probably also trapped on the hydrocarbon segments of the networks, which had a lower affinity toward CO_2_ molecules. The increased affinity of networks with CH=O and CH_2_OH towards CO_2_ is also evident from the comparison of the values of the isosteric heat of CO_2_ adsorption, *Q*_st_, determined for the coverage of the networks with CO_2_ of 10 mg/g. (Table 3). The *Q*_st_ values were calculated from the temperature dependences of CO_2_ adsorption isotherms. The *Q*_st_ value of P(pentyne/TEB 3:1) was 23 kJ/mol, while the *Q*_st_ values of the functionalized networks ranged from 26 to 29 kJ/mol and increased slightly with increasing content of CH=O and CH_2_OH groups in the networks. The high adsorption efficiency of networks functionalized with CH=O and CH_2_OH groups at a CO_2_ pressure in the range of 0–0.2 bar and the reversibility of CO_2_ adsorption fit the requirements for adsorbents suitable for the reversible capture of CO_2_ from gas mixtures with a low partial pressure of CO_2_, for example from flue gas [61].

### 3.3. Covalent Modification of Hydrolyzed Networks

**Scheme 3:**
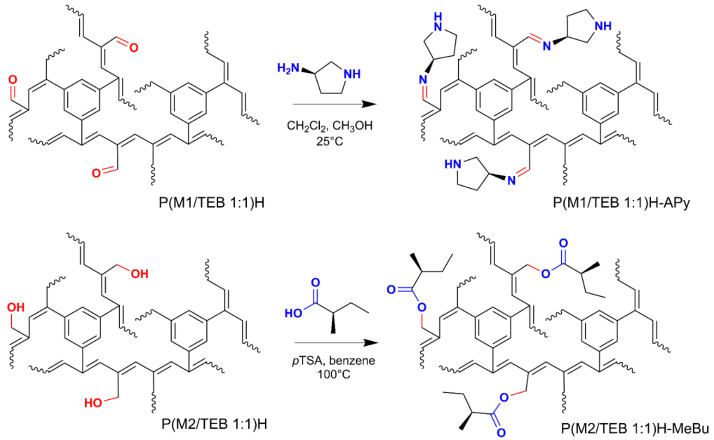
Postpolymerization covalent modification of the hydrolyzed networks P(M1/TEB 1:1)H and P(M2/TEB 1:1)H by chiral molecules of (*R*)-(+)-3-aminopyrrolidine and (*S*)-(+)-2-methylbutyric acid, respectively. Networks P(M1/TEB 1:1)H-APy and P(M2/TEB 1:1)H-MeBu were prepared.

The hydrolyzed networks discussed above represent porous supports whose surfaces could potentially be covalently modified with molecules of various functions. The covalent anchoring of such molecules could proceed via their reaction with reactive CH=O or CH_2_OH groups of the networks. We verified this possibility by modifying P(M1/TEB 1:1)H and P(M2/TEB 1:1)H networks with chiral molecules, (*R*)-(+)-3-aminopyrrolidine and (*S*)-(+)-2-methylbutyric acid, respectively (see Supplementary Materials for details). Networks P(M1/TEB 1:1)H and P(M2/TEB 1:1)H were selected for their high *S*_BET_ values (911 and 794 m^2^/g, respectively). The modification of P(M1/TEB 1:1)H with (*R*)-(+)-3-aminopyrrolidine proceeded through the imination reaction of the CH=O groups of the network with the NH_2_ groups of the modifying agent and resulted in the network labelled P(M1/TEB 1:1)H-APy in which the chiral pyrrolidine segments were attached to the surface via azomethine links (see Scheme 3). The modification of P(M2/TEB 1:1)H with (*S*)-(+)-2-methylbutyric acid proceeded through the esterification reaction between the OH groups of the network and the COOH groups of the modifying agent and resulted in the network labelled P(M2/TEB 1:1)H-MeBu in which the chiral 2-methylbutyric acid segments were attached to the surface via ester links (see Scheme 3). As expected, the covalent modification partially deteriorated the porosity of the networks: the *S*_BET_ values of modified P(M1/TEB 1:1)H-APy and P(M2/TEB 1:1)H-MeBu were 348 and 649 m^2^/g, respectively, i.e., from 20 to 60% lower than the *S*_BET_ of the networks before the modification (see Supplementary Materials, Scheme S2, Figures S8 and S9).

**Figure 7:**
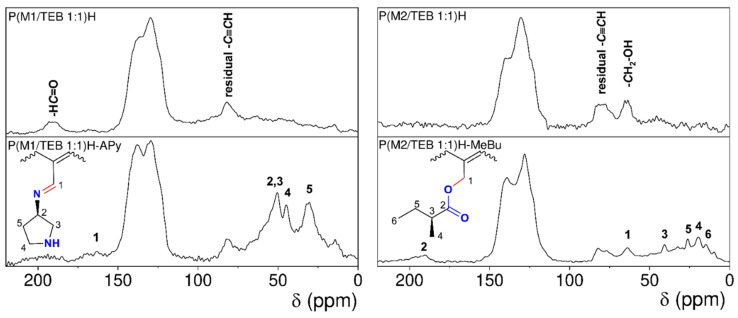
^13^C CP/MAS NMR spectra of the hydrolyzed networks P(M1/TEB 1:1)H and P(M2/TEB 1:1)H and modified networks P(M1/TEB 1:1)H-APy and P(M2/TEB 1:1)H-MeBu.

Figure 7 shows the ^13^C CP/MAS NMR spectra of the networks before and after covalent modification. The ^13^C CP/MAS NMR spectra of both modified networks P(M1/TEB 1:1)H-APy and P(M2/TEB 1:1)H-MeBu contained broad partly resolved signals in the aliphatic region, which we ascribed to the sp^3^ carbon atoms of pyrrolidine-3-yl and 2-methylbutyl segments, respectively (see Figure 7). The modification of P(M1/TEB 1:1)H to P(M1/TEB 1:1)H-APy was further accompanied by the disappearance of the signal of carbon atoms of CH=O groups (*δ* = 190 ppm) in the ^13^C CP/MAS NMR spectrum of the modified network. This signal was replaced by a weak signal at *δ* = 163 ppm corresponding to the resonance of the carbon atoms of the newly formed CH=N links. Similarly, the ^13^C CP/MAS NMR spectrum of P(M2/TEB 1:1)H-MeBu contained a signal at *δ* = 192 ppm due to the carbon atoms of the newly formed COO links. The extent of the modification of P(M1/TEB 1:1)H to P(M1/TEB 1:1)H-APy was about 75% (based on the ratio of N and C content in the modified network given by elemental analysis). In the modification of P(M2/TEB 1:1)H to P(M2/TEB 1:1)H-MeBu, about 40% of the CH_2_OH groups were modified by esterification with (*S*)-(+)-2-methylbutyric acid (based on the intensity of ^13^C CP/MAS NMR signals of P(M2/TEB 1:1)H-MeBu). Even if the above-discussed modifications were not quantitative, it was possible to introduce chiral groups into the networks in satisfactory amounts: 3.7 mmol/g in the case of P(M1/TEB 1:1)H-APy and 1.9 mmol/g in the case of P(M2/TEB 1:1)H-MeBu.

Evidently, the modification of the P(M1/TEB 1:1)H network via imination was more efficient than the esterification modification of P(M2/TEB 1:1)H. It should, however, be mentioned that modification of CH=O groups of P(M1/TEB 1:1)H via Schiff-base chemistry may be limited by the size of the molecules of the modifying agents. Our attempts to modify P(M1/TEB 1:1)H in reaction with dansylhydrazine (under formation of hydrazone links) failed (efficiency ˂ 10%), probably due to sterically hindered penetration of bulky dansylhydrazine molecules (C_12_H_15_N_3_O_2_S) into the pores. On the other hand, such a limitation could be useful for the size-controlled separation of mixtures of primary amines (hydrazines) through their chemisorption on P(M1/TEB 1:1)H or other CH=O groups containing POPs.

## 4. Conclusions

The chain-growth copolymerization of acetal-protected propargyl aldehyde and acetal-protected propargyl alcohol with 1,3,5-triethynylbenzene cross-linker followed by hydrolytic deprotection of the products provided hyper-cross-linked micro/mesoporous polyacetylene networks with CH=O and CH_2_OH groups attached without any spacer to the polyacetylene main chains of the networks. These networks can not be prepared by direct copolymerization of unprotected monomers due to the high reactivity of these monomers. The removable acetal segments not only protected the aldehyde and hydroxymethyl groups of the monomers during polymerization but also served as a template in the parent networks. The hydrolytic removal of these segments thus simultaneously modified the composition of the networks and their texture, which changed towards higher microporosity and higher specific surface area. The reported two-step synthesis thus allowed the preparation of micro/mesoporous networks with a tuneable content of CH=O and CH_2_OH groups (up to a high value of 9.6 mmol/g) and a specific surface area ranging from 400 to 800 m^2^/g.

The CH=O and CH_2_OH groups in the networks served as active centres for the physisorption of CO_2_ and water vapour. The networks were highly effective in reversible low-pressure CO_2_ adsorption. The networks also exhibited high water vapour capturing and releasing efficiency (capture capacity up to 445 mg/g at 297 K and RH = 90%), making them promising materials for water harvesting from the air. The water vapour capturing efficiency of the networks was controlled by the content of polar groups in the networks (low RH) and the total pore volume (high RH). Thanks to the presence of reactive groups CH=O and CH_2_OH, the networks represent supports suitable for the covalent anchoring of functional molecules, as shown by the modification of the networks with (*R*)-(+)-3-aminopyrrolidine and (*S*)-(+)-2-methylbutyric acid under formation of porous networks decorated with chiral moieties.

## Figures and Tables

**Table 1:** Specific surface area (*S*_BET_), micropore volume (*V*_mi_), total pore volume (*V*_tot_), and micropore diameter (*D*_mi_) (from N_2_ adsorption isotherms) of prepared networks.

Network Code	*S_BET_* (m^2^/g)	*V*_mi_ (cm^3^/g)	*V*_tot_ (cm^3^/g)	*D*_mi_ (nm)
P(M1/TEB 1:1)	832	0.29	0.90	0.9
P(M1/TEB 1:1)H	911	0.35	0.60	0.7
P(M1/TEB 3:1)	313	0.12	0.19	1.1
P(M1/TEB 3:1)H	534	0.22	0.28	0.7
P(M2/TEB 1:1)	393	0.13	0.71	1
P(M2/TEB 1:1)H	794	0.29	0.76	0.7
P(M2/TEB 3:1)	149	0.05	0.11	1.1
P(M2/TEB 3:1)H	409	0.17	0.22	0.7

**Table 2:** H_2_O adsorption capacities *a*_H2ORH40_ (at RH = 40%) and *a*_H2ORH90_ (at RH = 90%) of the networks at 297 K. *V*_tot_ values were obtained from N_2_ adsorption isotherms at 77 K.

Network Code	Polar Group Content (mmol/g)	*V*_tot_ (cm^3^/g)	*a*_H2ORH40_ (mg/g)	*a*_H2ORH90_ (mg/g)
P(M1/TEB 1:1)H	4.90	0.60	100	445
P(M1/TEB 3:1)H	9.61	0.28	139	272
P(M2/TEB 1:1)H	4.85	0.76	56	422
P(M2/TEB 3:1)H	9.42	0.22	98	286
P(pentyne/TEB 3:1)	0	1.69	14	36

**Table 3:** CO_2_ adsorption capacities of the networks at an equilibrium CO_2_ pressure of 0.2 and 1 bar at 273.15 K (*a*_CO2_ and *a*_CO2/S_) and isosteric heats of the CO_2_ adsorption, *Q*_st_, at the coverage of 10 mg(CO_2_)/g. *S*_BET_ values were obtained from N_2_ adsorption isotherms at 77 K.

Network Code	Polar Group Content (mmol/g)	*S*_BET_ (m^2^/g)	0.2 bar		1 bar		*Q*_st_ (kJ/mol)
			*a*_CO2_ (mg/g)	*a*_CO2/*S*_ (µg/m^2^)	*a*_CO2_ (mg/g)	*a*_CO2/*S*_ (µg/m^2^)	
P(M1/TEB 1:1)H	4.90	911	38.1	41.8	99.0	108.7	26
P(M1/TEB 3:1)H	9.61	534	42.2	79.0	87.6	164.0	29
P(M2/TEB 1:1)H	4.85	749	26.7	35.6	72.6	96.9	26
P(M2/TEB 3:1)H	9.42	409	32.9	80.4	75.2	184.0	27
P(pentyne/TEB 3:1)	0	810	13.5	16.7	39.2	48.3	23

## Data Availability

The data presented in this study are available on request from the corresponding author.

## Supplementary Materials

The following supporting information can be downloaded at: https://www.mdpi.com/article/10.3390/polym15030743/s1, Figure S1: Thermogravimetric analysis of the networks; Scheme S1: Copolymerization of 1-pentyne with triethynylbenzene (TEB) resulting in P(pentyne/TEB 3:1); Table S1: The textural parameters of the P(pentyne/TEB 3:1) network; Figure S2: ^13^C CP/MAS NMR spectrum of P(pentyne/TEB 3:1); Figure S3: N_2_ adsorption and desorption isotherms (77 K) on P(pentyne/TEB 3:1); Figure S4: Micropore size distribution of P(pentyne/TEB 3:1); Figure S5: The time course of adsorption/desorption of water on the networks (297 K) reported as a change in mass vs. time.; Figure S6: H_2_O adsorption and desorption isotherms on the networks at 297 K. Two consecutive measurements; Figure S7: CO_2_ adsorption and desorption on the networks at 273, 293, 313, and 333 K; Scheme S2: Postpolymerization chemisorption modification of the hydrolyzed networks P(M1/TEB 1:1)H and P(M2/TEB 1:1)H by chiral molecules of (*R*)-(+)-3-aminopyrrolidine and (*S*)-(+)-2-methylbutyric acid.; Figure S8: N_2_ adsorption and desorption isotherms (77 K) on the networks P(M1/TEB 1:1)H, P(M2/TEB 1:1)H, P(M1/TEB 1:1)H-Apy, and P(M2/TEB 1:1)H-MeBu; Figure S9: Changes in micropore size distributions due to the modification of the networks with chiral moieties.
