# Supplementary material for: Combining Polymerization and Templating toward Hyper-Cross-Linked Poly(propargyl aldehyde)s and Poly(propargyl alcohol)s for Reversible H2O and CO2 Capture and Construction of Porous Chiral Networks
Source: Polymers (Basel). 2023 Feb 1;15(3):743. doi: 10.3390/polym15030743 (PMC9919244; doi:10.3390/polym15030743)
Supplement: Supplementary file 1 [file polymers-15-00743-s001.zip › polymers-2175579-supplementary.pdf]

Supplementary Materials

Combining polymerization and templating toward hyper-cross-linked poly(propargyl aldehyde)s and poly(propargyl alcohol)s for reversible H_2O and CO_2 capture and construction of porous chiral networks

Lucie Havelková [1,], Bogdana Bashta [1], Alena Hašková [1], Alice Vagenknechtová [2], Eliška Vyskočilová [3], Jiří Brus [4] and Jan Sedláček [1,]**

1 Department of Physical and Macromolecular Chemistry, Faculty of Science, Charles University, Hlavova 2030, 12843, Prague, Czech Republic

2 Department of Gaseous and Solid Fuels and Air Protection, University of Chemistry and Technology in Prague, Technická 5, Prague, 166 28 Czech Republic

3 Department of Organic Technology, University of Chemistry and Technology in Prague, Technická 5, Prague, 166 28 Czech Republic

4 Institute of Macromolecular Chemistry, Czech Academy of Sciences, Heyrovský Sq.2, Prague, 162 00, Czech Republic

* Correspondence: lucie.havelkova@natur.cuni.cz (L.H.); jan.sedlacek@natur.cuni.cz (J.S.)

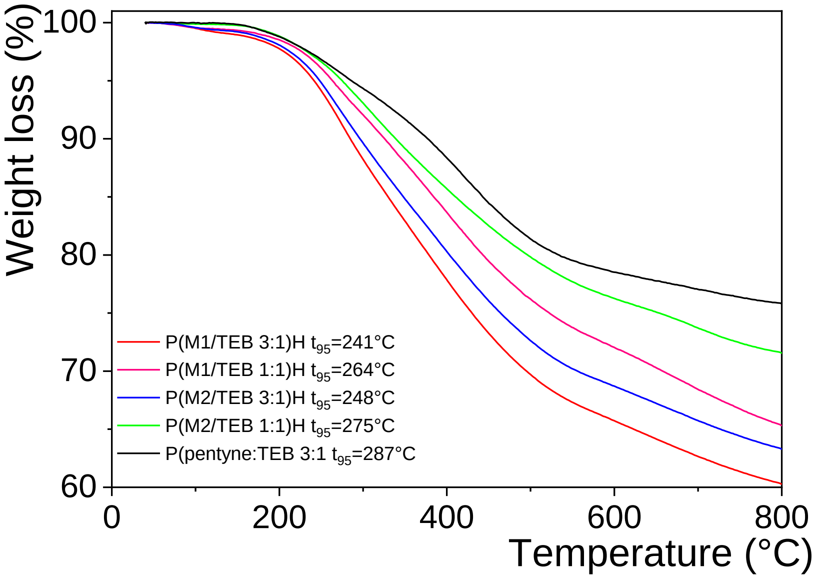

Figure S1. Thermogravimetric analysis of the networks.

Synthesis of P(pentyn/TEB 3:1)

Copolymerization of 1-pentyne with 1,3,5-triethynylbenzene (TEB) was performed in CH_2Cl_2 at 75 °C in a sealed thick-wall ampoule under an argon atmosphere using the [Rh(nbd)acac] complex as a polymerization catalyst. The reaction time was 7 days. The initial concentration of comonomers was 0,3 mol/dm^3 and the concentration of [Rh(nbd)acac] was 15 mmol/dm^3. The comonomer mole ratio in the feed 1-pentyne:TEB was 3:1. 600 mg of 1-pentyne and 441 mg of TEB were dissolved in 35 ml of CH_2Cl_2. A solution of 173 mg of the catalyst in 4 ml of CH_2Cl_2 was added to the solution of comonomers to start the polymerization. After 7 days, the precipitated polymer was separated, repeatedly washed with CH_2Cl_2, and dried under vacuum for 2 days at room temperature. The polymer network P(pentyne/TEB 3:1) was formed in quantitative yield.

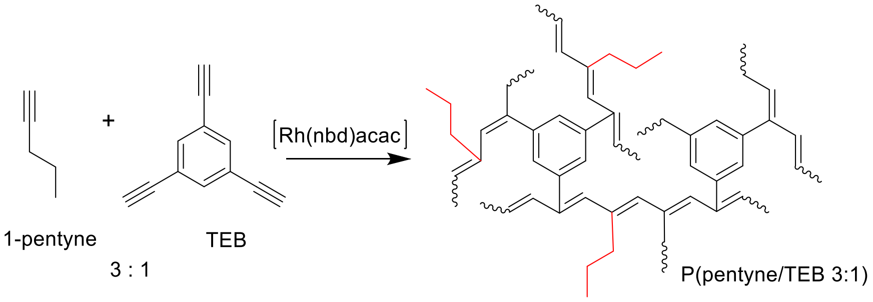

Scheme S1. Copolymerization of 1-pentyne with triethynylbenzene (TEB) resulting in P(pentyne/TEB 3:1).

Table S1. The textural parameters of the P(pentyne/TEB 3:1) network.

Network code	S_{BET} [m^2/g]	V_{mi} [cm^3/g]	V_{tot} [cm^3/g]	D_{mi} [nm]
P(pentyne/TEB 3:1)	810	0.28	1.69	0.9

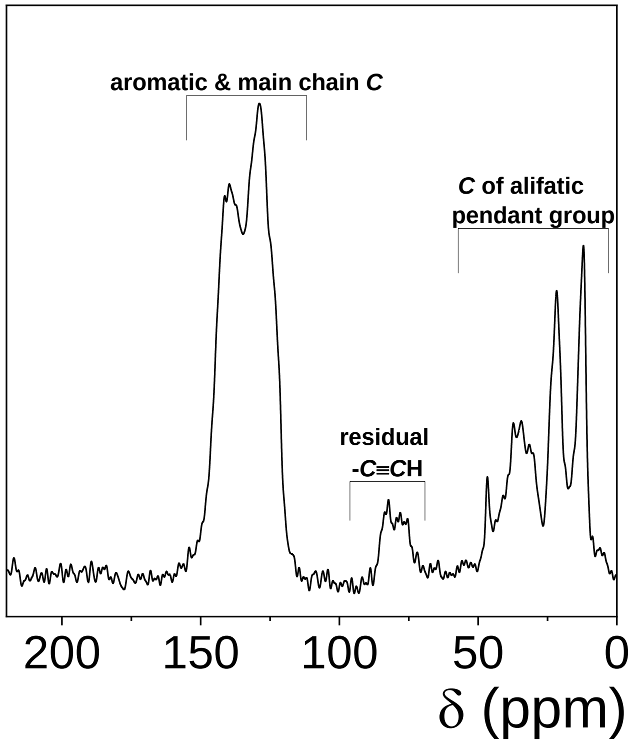

Figure S2. ^{13}C CP/MAS NMR spectrum of P(pentyne/TEB 3:1).

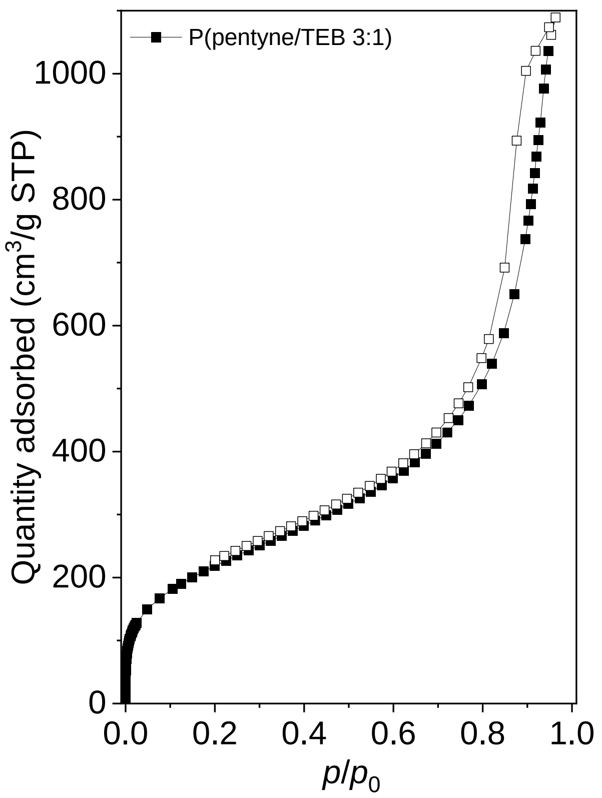

Figure S3. N_2 adsorption (full points) and desorption (empty points) isotherms (77 K) on P(pentyne/TEB 3:1).

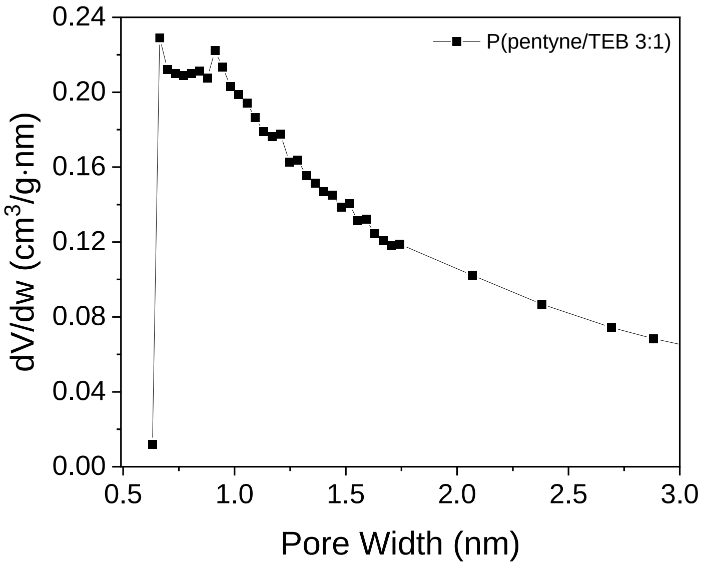

Figure S4. Micropore size distribution of P(pentyne/TEB 3:1).

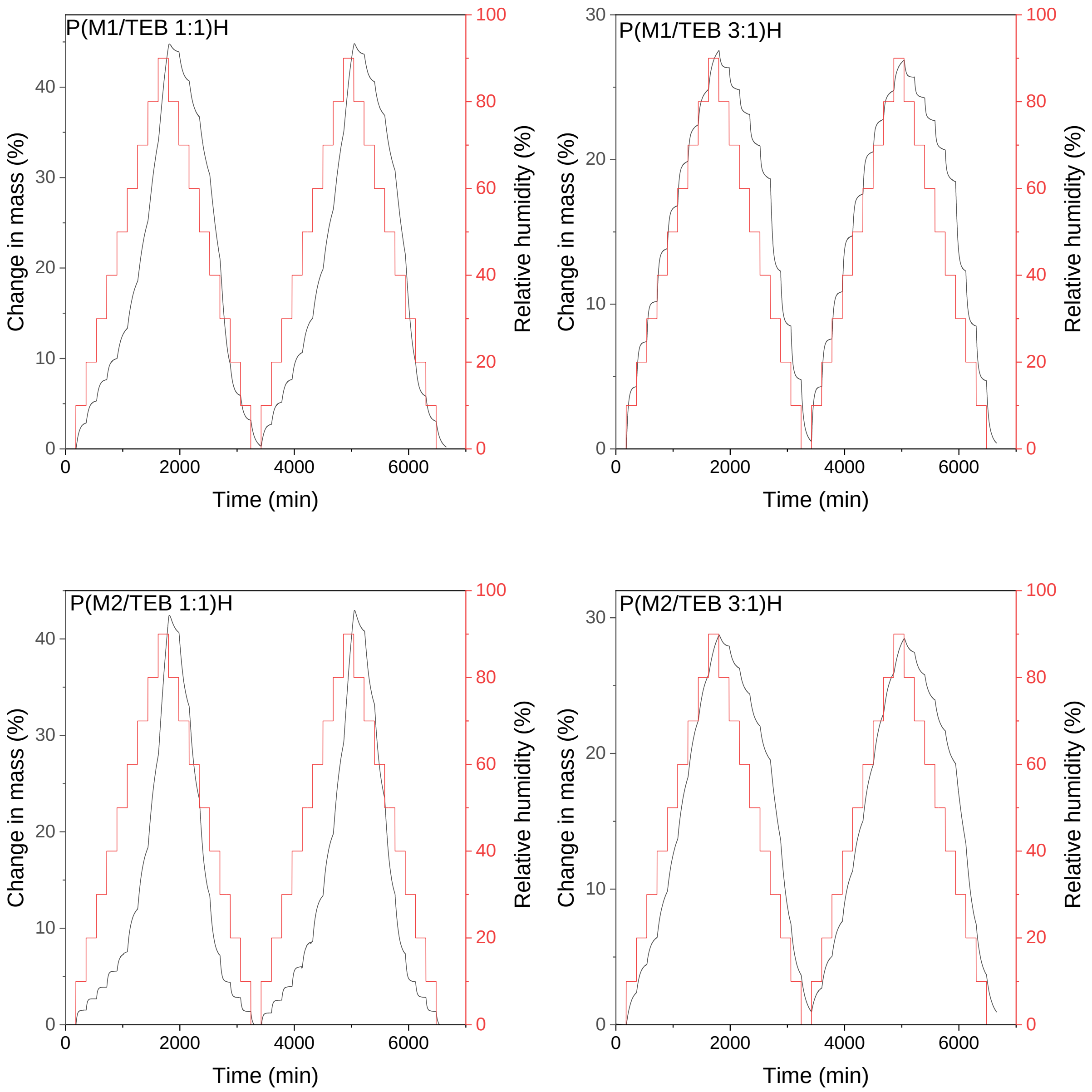

Figure S5. The time course of adsorption/desorption of water on the networks (297 K) reported as a change in mass vs. time (black line). Relative humidity of the gas phase (red line).

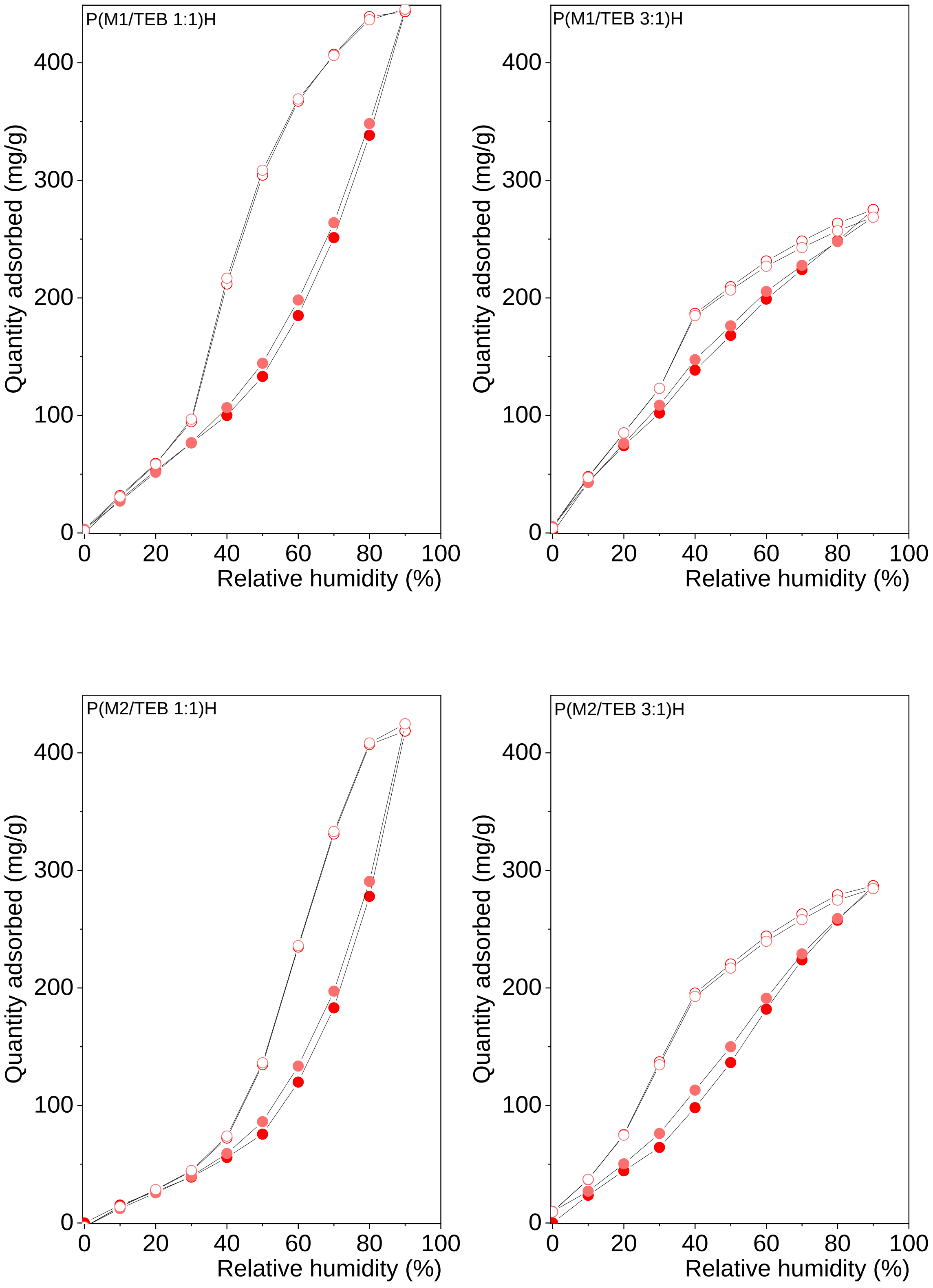

Figure S6. H_2O adsorption (full points) and desorption (empty points) isotherms on the networks at 297 K. Two consecutive measurements.

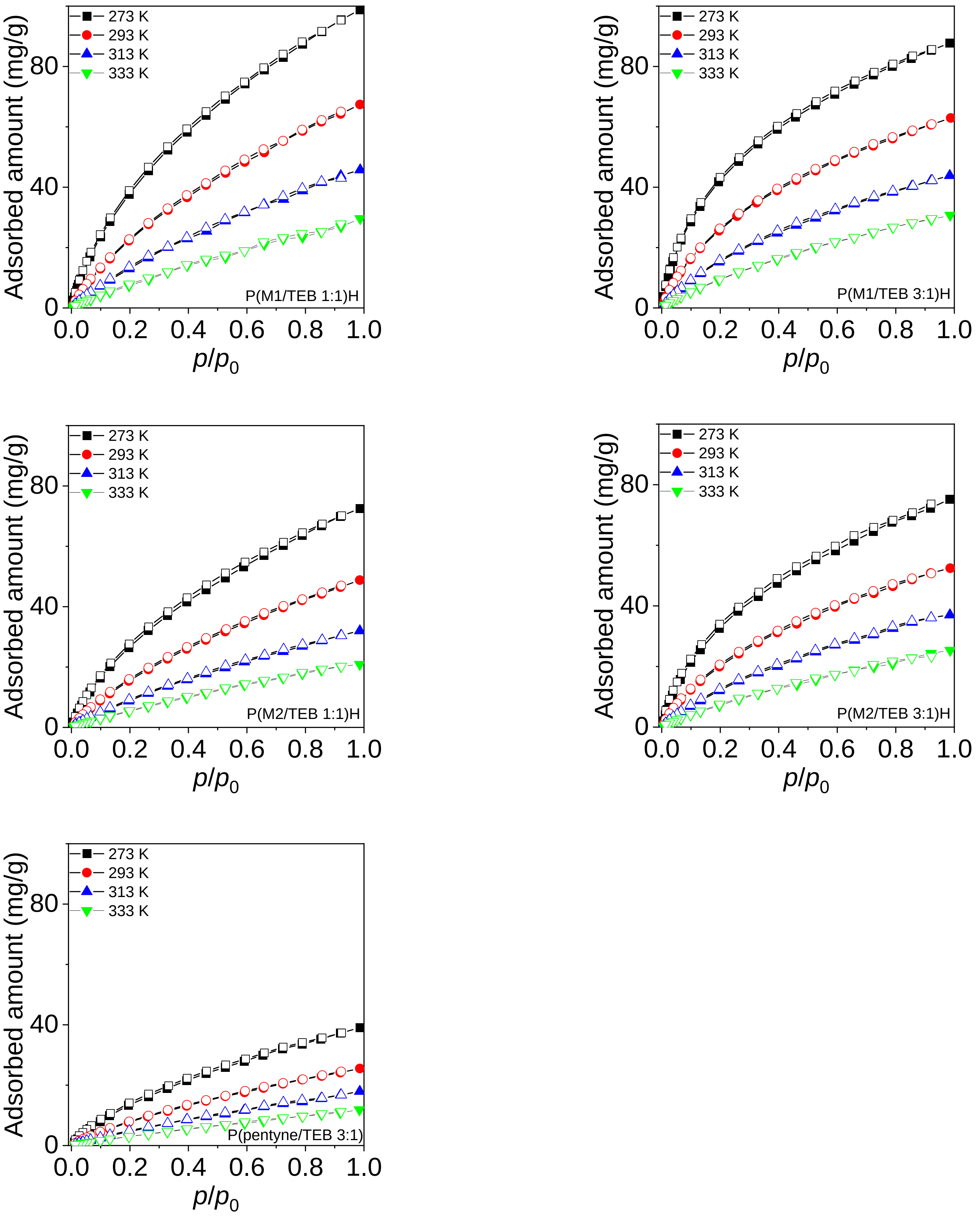

Figure S7. CO_2 adsorption (full points) and desorption (empty points) on the networks at 273, 293, 313 and 333 K.

Postpolymerization modification of P(M1/TEB 1:1)H with (*R*)-(+)-3-aminopyrrolidine

The postpolymerization covalent modification of P(M1/TEB 1:1)H with (*R*)-(+)-3-aminopyrrolidine was performed in the mixture of dichloromethane and methanol (2/1; v/v) at room temperature under stirring. The reaction time was 14 days. In a typical experiment, 180 mg of P(M1/TEB 1:1)H was reacted with 385 mg of (*R*)-(+)-3-aminopyrrolidine. An excess of modifier (NH_2 groups to CHO groups mole ratio = 5) was used to support the modification of CHO groups. The P(M1/TEB 1:1)H network covalently modified with (*R*)-(+)-3-aminopyrrolidine was separated by filtration, repeatedly washed with methanol and dichloromethane to remove physisorbed amine and dried under vacuum at room temperature to constant weight.

Postpolymerization modification of P(M2/TEB 1:1)H with (*S*)-(+)-2-methylbutyric acid

The postpolymerization covalent modification of P(M2/TEB 1:1)H with (*S*)-(+)-2-methylbutyric acid was performed as azeotropic esterification in benzene. In a typical experiment, 500 mg of (*S*)-(+)-2-methylbutyric acid was dissolved in 50 ml of benzene and 23 mg of *p*-toluenesulfonic acid was added and dissolved, then 200 mg of P(M2/TEB 1:1)H was dispersed in this solution. An excess of modifier (COOH groups to OH groups mole ratio = 5) was used to support the modification of OH groups. The mixture was stirred at 100°C for 4 days. The reaction was terminated by adding the excess of benzene (200 ml). The P(M2/TEB 1:1)H network covalently modified with (*S*)-(+)-2-methylbutyric acid was separated by filtration and repeatedly washed with benzene to remove physisorbed carboxylic acid and dried under vacuum at room temperature to constant weight.

Chemisorption of dansyl hydrazine on P(M1/TEB 1:1)H

The chemisorption was performed in the mixture of dichloromethane and methanol (4/1; v/v) at room temperature under stirring. In a typical experiment, 425 mg of dansyl hydrazine (DH) was dissolved in the mixture of 4 ml dichloromethane and 1 ml of methanol and then 100 mg of P(M1/TEB 1:1)H was dispersed in the solution. An excess of chemisorbent (NH_2 groups to CHO groups mole ratio = 3 for DH) was used to achieve maximum chemisorption capacity of P(M1/TEB 1:1)H. After 14 days, P(M1/TEB 1:1)H with chemisorbed DH was repeatedly washed with the mixture of dichloromethane and methanol (4/1; v/v) to removed physisorbed DH, then separated by filtration and dried under vacuum at room temperature to constant weight.

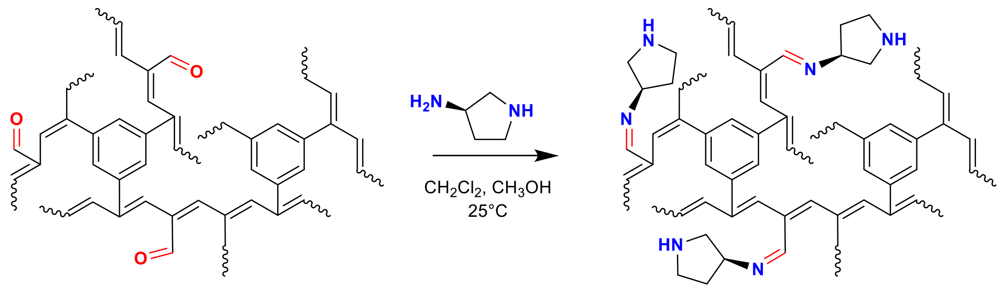

P(M1/TEB 1:1)H

S_{BET}=911 m^2/g; V_{mi}=0.35 cm^3/g; V_{tot}=0.6 cm^3/g; D_{mi}=0.7 nm

P(M1/TEB 1:1)H-APy

S_{BET}=348 m^2/g; V_{mi}=0.12 cm^3/g; V_{tot}=0.27 cm^3/g; D_{mi}=0.9 nm

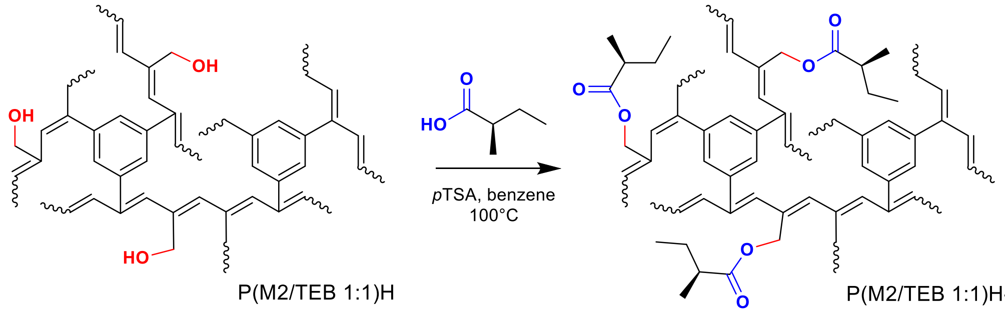

P(M2/TEB 1:1)H

S_{BET}=794 m^2/g; V_{mi}=0.29 cm^3/g; V_{tot}=0.76 cm^3/g; D_{mi}=0.7 nm

P(M2/TEB 1:1)H-MeBu

S_{BET}=649 m^2/g; V_{mi}=0.24 cm^3/g; V_{tot}=0.68 cm^3/g; D_{mi}=0.7 nm

Scheme S2. Postpolymerization chemisorption modification of the hydrolyzed networks P(M1/TEB 1:1)H and P(M2/TEB 1:1)H by chiral molecules of (R)-(+)-3-aminopyrrolidine and (S)-(+)-2-methylbutyric acid, respectively. Networks P(M1/TEB 1:1)H-APy and P(M2/TEB 1:1)H-MeBu were prepared.

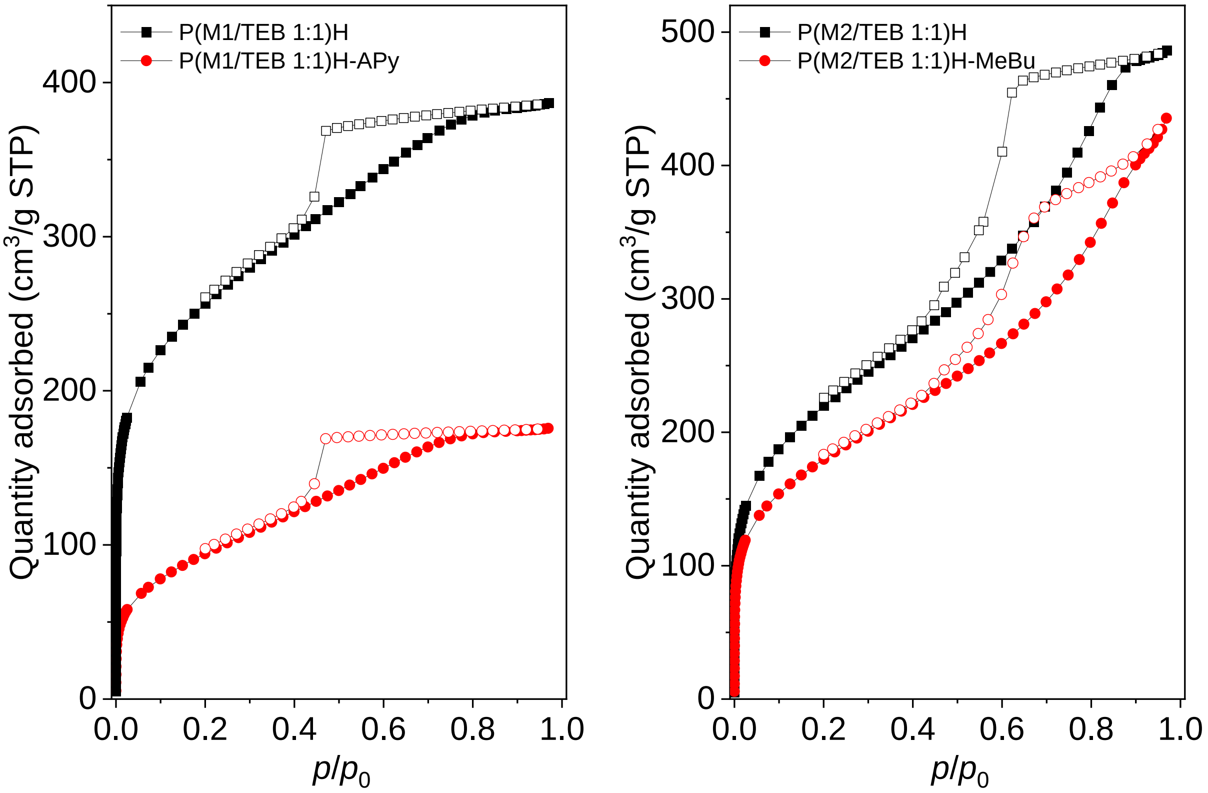

Figure S8. N_2 adsorption (full points) and desorption (empty points) isotherms (77 K) on the networks P(M1/TEB 1:1)H, P(M2/TEB 1:1)H, P(M1/TEB 1:1)H-APy and P(M2/TEB 1:1)H-MeBu.

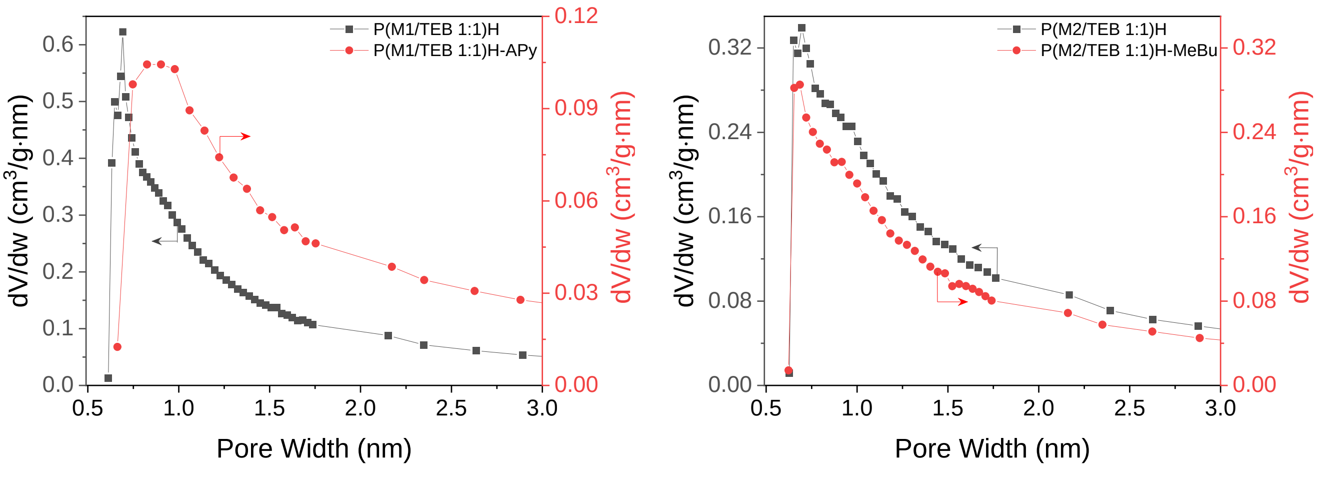

Figure S9. Changes in micropore size distributions due to the modification of the networks with chiral moieties.
